# Culturing of *Giardia lamblia* under microaerobic conditions can impact metronidazole susceptibility by inducing increased expression of antioxidant enzymes

**DOI:** 10.1016/j.ijpddr.2025.100585

**Published:** 2025-02-01

**Authors:** Kateryna Starynets, Ana Paunkov, Anja Wagner, Klaus Kratochwill, Christian Klotz, David Leitsch

**Affiliations:** a Institute for Specific Prophylaxis and Tropical Medicine, Center for Pathophysiology, Infectiology, and Immunology, Medical University of Vienna, Kinderspitalgasse 15, A-1090, Vienna, Austria; b Core Facility Proteomics, Medical University of Vienna, A-1090, Vienna, Austria; c Division of Pediatric Nephrology and Gastroenterology, Department of Pediatrics and Adolescent Medicine, Comprehensive Center for Pediatrics, Medical University of Vienna, A-1090, Vienna, Austria; d Department of Infectious Diseases, Unit 16 Mycotic and Parasitic Agents and Mycobacteria, Robert Koch-Institute, Berlin, Germany

## Abstract

The microaerophilic/anaerobic protist *Giardia lamblia* is a world-wide occurring parasite of the human small intestine. It causes giardiasis which manifests as diarrhoea accompanied by other sequelae. Giardiasis is most commonly treated with either the 5-nitroimidazole metronidazole or the benzimidazole albendazole. Unfortunately, the number of refractory cases is increasing, which is probably caused, at least in part, by drug resistance. However, most attempts to isolate metronidazole-resistant *G*. *lamblia* strains from patients have failed so far because the parasites were not resistant when tested *in vitro*.

We hypothesized that this failure might be caused by drug assay conditions which are standardly anaerobic, and performed metronidazole susceptibility testing with two well studied strains, i.e. WB C6 and BRIS/87/HEPU/713 (strain 713) under microaerophilic conditions. Indeed, 713 proved to be less susceptible to metronidazole under microaerophilic conditions as compared to anaerobic conditions, and residual growth was even noted at concentrations of metronidazole similar to those in the serum of treated patients (i.e. about 100 μM). Further experiments showed that 713 also grows much faster under microaerobic conditions than WB C6. Reduced susceptibility to metronidazole under microaerobic conditions was also observed in a clinical isolate from a refractory giardiasis case.

Two-dimensional gel electrophoresis showed that microaerobic growth was accompanied by the upregulation of superoxide reductase, a pyridoxamine 5′-phosphate oxidase putative domain-containing protein, and a TlpA-like protein in 713 but not in WB C6. All three proteins are known, or can be predicted to have antioxidant functions. Indeed, overexpression of pyridoxamine 5′-phosphate oxidase in WB C6 from a plasmid carrying the respective gene behind the arginine deiminase promoter significantly improved growth of the transfected cell line under microaerobic conditions. Moreover, similarly overexpressed superoxide reductase conferred significant protection against metronidazole.

Our results suggest that oxygen concentrations can affect the outcomes of metronidazole treatment against *G*. *lamblia*.

## Highlights

• *G. lamblia* is less susceptible to metronidazole under microaerobic conditions.
• In strain 713 this leads to borderline resistance.
• Strain 713 displays an overall better growth under microaerobic conditions.
• In presence of oxygen certain antioxidant factors are overexpressed in 713.
• Superoxide reductase protects against metronidazole.

## Graphical abstract

**Figure:**
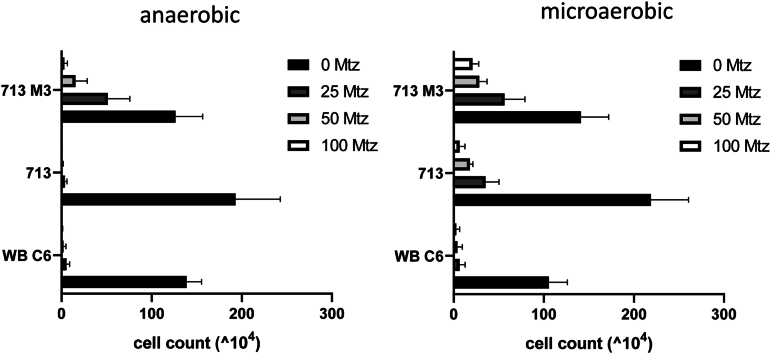

## 1 Introduction

The microaerophilic/anaerobic protist *Giardia lamblia* (syn: *intestinalis*, *duodenalis*) is the causative agent of giardiasis, an infection of the small intestine in humans. Giardiasis is characterized by diarrhoea and other accompanying gastrointestinal symptoms, and can persist for weeks or even months (Adam, 2021). In children, giardiasis can trigger stunting or cause failure to thrive (Adam, 2021). Since a vaccine is not available against *G*. *lamblia*, management of giardiasis is restricted to the chemotherapy of infected individuals. The 5-nitroimidazole metronidazole and the benzimidazole albendazole are by far the most frequently prescribed drugs in the treatment of giardiasis (Argüello-García et al., 2020). Although metronidazole was originally highly effective, reports on refractory cases are mounting, especially in some parts of the world (Carter et al., 2018). The observed increase is commonly attributed to a rise in metronidazole resistance, but to best of our knowledge metronidazole resistance in *G*. *lamblia* clinical strains could be confirmed only once after isolation and testing *in vitro* (Lemée et al., 2000). Indeed, all metronidazole-resistant cell lines of *G*. *lamblia* strains studied so far were generated *in vitro* (Argüello-García et al., 2020). This is reminiscent of the situation in *Trichomonas vaginalis*, another microaerophilic human parasite, before the discovery of aerobic metronidazole susceptibility testing (Meingassner and Thurner, 1979). Metronidazole resistance in clinical *T*. *vaginalis* isolates only becomes manifest when susceptibility testing is performed in the presence of oxygen. This is highly relevant because *T*. *vaginalis* and *G*. *lamblia* do not inhabit strictly anaerobic body niches but the urogenital tract and the small intestine, respectively, where oxygen is present, albeit at lower concentrations. However, aerobic drug susceptibility testing as performed with *T*. *vaginalis* is not feasible with *G*. *lamblia* because it does not endure high oxygen concentrations for prolonged periods of time (Gillin and Diamond, 1981).

In the search of a remedy to this problem we aimed at defining test conditions which are more similar to the environment as provided by the host. To this end we applied a microaerophilic atmosphere generating system, CampyGen, which was developed specifically for growing *Campylobacter jejuni*, a microaerophilic bacterium inhabiting the same body niche as *G*. *lamblia*. Importantly, CampyGen has been recently introduced to metronidazole susceptibility testing in *T*. *vaginalis* and *Tritrichomonas foetus* (Lam et al., 2023). We performed metronidazole susceptibility testing in parallel under anaerobic and microaerophilic conditions with several *G*. *lamblia* strains, including the widely used laboratory strains WB C6 and BRIS/87/HEPU/713 (commonly abbreviated to 713), and compared the results obtained.

## 2 Materials and methods

### 2.1 Materials

Metronidazole was purchased from Sigma-Aldrich (Merck Life Science, Darmstadt, Germany). CampyGen 2.5L and AnaeroGen 2.5L atmosphere generation sachets were purchased from Thermo Fisher Scientific (Waltham, USA), and Anaerocult A sachets were purchased from Merck. The components of Keister's modified TYI-S-33 *Giardia* growth medium (Keister, 1983) were purchased from Merck (peptone, yeast extract, sodium chloride, potassium dihydrogen phosphate) and from Sigma-Aldrich (glucose, cysteine, ascorbic acid, foetal bovine serum, bovine bile).

### 2.2 Strains

Strain WB C6 (or ATCC 50803) is a derivative of the W.B. isolate (Smith et al., 1982) and strain BRIS/87/HEPU/713 originates from the strain collection of the Queensland Institute of Medical Research (QIMR), Australia (Capon et al., 1989). Henceforth, BRIS/87/HEPU/713 will be abbreviated to 713 throughout the manuscript. Strain 713-M3 is a metronidazole-resistant cell line of 713 generated *in vitro* (Townson et al., 1992). The clinical isolates P064-F7 (henceforth abbreviated to P064), assemblage AII, and P344-B2 (henceforth abbreviated to P344), assemblage BIII, are from a *Giardia* strain collection established by Christian Klotz between 2011 and 2015 at the Robert Koch-Institute in Berlin (Klotz et al., 2023). Both clinical strains are grown in Keister's *Giardia* growth medium with foetal bovine serum substituted for adult bovine serum.

### 2.3 Cell culture

Cells were routinely grown in Keister's modified TYI-S-33 medium (Keister, 1983) in Nunc™ cell culture tubes (Thermo Fisher Scientific).

### 2.4 Susceptibility assays and growth assays

Susceptibility assays were performed in 24 well plates filled with 2 mL of growth medium per well because cells grew poorly when 96 well plates were used. Metronidazole was added in appropriate concentrations. Subsequently, wells were inoculated with 1 × 10^5^ cells each and plates were incubated at 37 °C for 48 h in 2.5 L air-tight plastic boxes together with CampyGen (Lam et al., 2023) to generate microaerobic conditions (8–9% oxygen, 7–8% carbon dioxide), or with Anaerocult A or AnaeroGen to generate anaerobic conditions. Anaerocult A was preferably used because we had used it in earlier studies (Mayr et al., 2024), but if it was commercially unavailable AnaeroGen was used instead. After 48 h, live cells in the wells were counted in a Bürker-Türk counting chamber under a microscope. Only motile cells were interpreted as live cells. Susceptibility assays for the determination of the IC_50_ of metronidazole were also performed in 24 well plates but with inoculums of 4 × 10^4^ cells and with eight different metronidazole concentrations applied, i.e. 0, 1, 2, 5, 10, 20, 50, and 100 μM in WB C6, 713, and P064; and 0, 5, 10, 20, 50, 100, 200, and 300 μM in 713-M3. Figures were generated using GraphPad Prism 10 software. If not indicated otherwise, the non-linear regression curves were calculated using the following settings: “Absolute IC50, X is concentration”.

Growth assays were performed with inoculums of 2 × 10^4^ cells per well but otherwise as described above for metronidazole susceptibility assays.

### 2.5 Two-dimensional gel electrophoresis (2DE)

2D gel electrophoresis was performed as previously described (Leitsch et al., 2005, 2011). Briefly, 400 μg of protein were solubilized in 2DE sample buffer (7 M urea, 2 M thiorea, 1% DTT, 4% CHAPS, 1% ampholytes) and loaded on 17 cm immobilized pH gradient (IPG) strips in the pH range of 3–10, non-linear gradient (Bio-Rad Laboratories, Hercules, CA, USA). Isolelectric focusing was performed in a Protean IEF cell (Bio-Rad): rehydration (50 V, 12 h), 150 V (rapid slope, 1 h), 300 V (rapid slope, 1 h), 2000 V (linear slope, 1 h), 5000 V (linear slope, 2 h), 10,000 V (rapid slope, 7 h). Afterwards strips were equilibrated for gel electrophoresis and SDS-PAGE (12.5% acrylamide; 0.32% bisacrylamide) was performed in Protean II Xi cells (Bio-Rad). Gels were stained with Coomassie Brilliant blue R250 (Sigma) and densitometric analyses were performed with Delta2D software (Decodon, Greifswald, Germany).

### 2.6 Mass-spectrometric identification of isolated proteins

Protein spots of interest were cut out from Coomassie-stained 2D-gels and tryptic in gel-digestion of the excised spots was performed according to the protocol of Shevchenko (Shevchenko et al., 1996) with slight modifications. The plugs were washed using H_2_O_UHQ_ (VWR), 100 mM NH_4_HCO_3_, 100 mM NH_4_HCO_3_/ethanol (VWR, 50:50) and acetonitrile (ACN, Merck) until the plugs were fully destained. After a reduction step with 10 mM dithiothreitol (DTT) in 25 mM NH_4_HCO_3_ for 1 h at 56 °C, the samples were alkylated using 55 mM iodoacetamide (IAA) in 25 mM NH_4_HCO_3_ for 45 min at 25 °C in the dark. Then the plugs were washed again with 100 mM NH_4_HCO_3_ and ACN before adding a 0.59 μg trypsin (Promega) in 50 mM NH_4_HCO_3_ for digestion overnight at 37 °C. The cleaved peptides were eluted from the plugs with a mixture of ACN/H_2_O_UHQ_/trifluoroacetic acid (TFA) (50:45:5) and sonication. Afterwards the eluates were dried by vacuum centrifugation and the peptides were reconstituted in 0.1% TFA. Stage Tips were prepared by placing small discs of C18 reversed-phase beads embedded in Teflon mesh (Empore Disks, Sigma) into pipette tips using a metal needle. The membrane was wetted with methanol (VWR) and washed with 0.4% formic acid (FA, Merck), 2% TFA. After loading of the sample, the membrane was washed again with 0.4% (FA), 2% TFA followed by elution with 90% ACN, 0.4% FA. Afterwards the eluates were dried by vacuum centrifugation and the peptides were reconstituted in 0.1% TFA.

Samples were analyzed on an Ultimate 3000 RSLC nano coupled directly to an Exploris 480 with FAIMSpro (all Thermo Fisher Scientiﬁc). Samples were injected onto a reversed-phase C18 column (50 cm × 75 μm i.d., packed in-house) and eluted with a gradient of 4%–38% mobile phase B (90% ACN, 0.4% FA) over 94 min by applying a flow rate of 230 nL/min. MS scans were performed in the range from *m*/*z* 375–1650 at a resolution of 60,000 (at *m*/*z* = 200). MS/MS scans were performed choosing a resolution of 15,000; normalized collision energy of 29%; isolation width of 1.4 *m/z* and dynamic exclusion of 90s. Two different FAIMS voltages were applied (−40 V and −60 V) with a cycle time of 1.5 s per voltage. FAIMS was operated in standard resolution mode with a static carrier gas flow of 4.1 L/min.

The acquired raw MS data files were processed and analyzed using ProteomeDiscoverer (v2.4.0.305, Thermo Fisher Scientiﬁc). SequestHT was used as search engine and following parameters were chosen: database: *Giardia lamblia* (UniProt, organism ID 5741, downloaded on 2023-12-07); enzyme: trypsin; max. missed cleavage sites: 2; static modifications: carbamidomethyl (C); dynamic modifications: oxidation (M), acetyl (protein N-terminus), Met-loss (M) and Met-loss + Acetyl (M); precursor mass tolerance: 10 ppm; fragment mass tolerance: 0.02 Da.

### 2.7 Overexpression of candidate enzymes in WB C6

The genes for superoxide reductase (NCBI: XM_038046279), pyridoxamine 5′-phosphate oxidase putative domain-containing protein (NCBI: XM_001705540), and TlpA-like family protein (NCBI: XM_001707479) were amplified from genomic DNA isolated from WB C6 (ATCC 50803). The respective forward primers included 50 bp of the upstream region of either the arginine deiminase gene (XM_001705703) for pyridoxamine 5′-phosphate oxidase and TlpA, or of the glutamate dehydrogenase gene for superoxide reductase. Both upstream regions contain the promoters of the respective genes (Leitsch et al., 2016; Müller et al., 2009). Primer sequences are given in Supplementary Table 1. PCR products were digested with *Pac*I and *Xba*I and cloned into the pPac-VInteg vector (Štefanić et al., 2009) and the resulting plasmids were propagated in *E*. *coli* 10-beta (New England Biolabs). WB C6 trophozoites were transfected in a BTX Electro cell manipulator 600 (Harvard Apparatus) applying the following settings: 500 V, 800 mF, and 720 Ω. Transfected trophozoites were subsequently selected with puromycin (100 μg/mL).

### 2.8 RT-qPCR

RNA was isolated from cells of overnight cultures with the GeneJet RNA Purification Kit (Thermo Fisher Scientific). Subsequently, the isolated RNA was diluted to a concentration of 10 ng/μL and used for RT-qPCR applying the Luna Universal One-Step RT-qPCR kit (New England Biolabs). Reactions were performed in 96-well plates using a CFX Connect Real-Time System (Bio-Rad). Concentrations of the mRNAs studied were determined in at least three biological experiments in technical triplicates. Relative mRNA concentrations were determined by normalization against glutamate dehydrogenase mRNA (*gdh*) (Müller et al., 2009) and glyceraldehyde-3-phosphate dehydrogenase mRNA (*gapdh*) (Barash et al., 2017) as internal standards. The calculation of primer efficiencies and mRNA concentrations was performed as according to Vandesompele et al. (2002); Hellemans et al. (2007). Primer sequences are given in Supplementary Table 1.

### 2.9 Statistical analysis

All statistical tests were performed using GraphPad Prism 10 software. Details are given in the respective figure legends.

## 3 Results

### 3.1 Determination of metronidazole susceptibility in WB C6, 713, and 713-M3 under anaerobic and microaerobic conditions

**Fig. 1:**
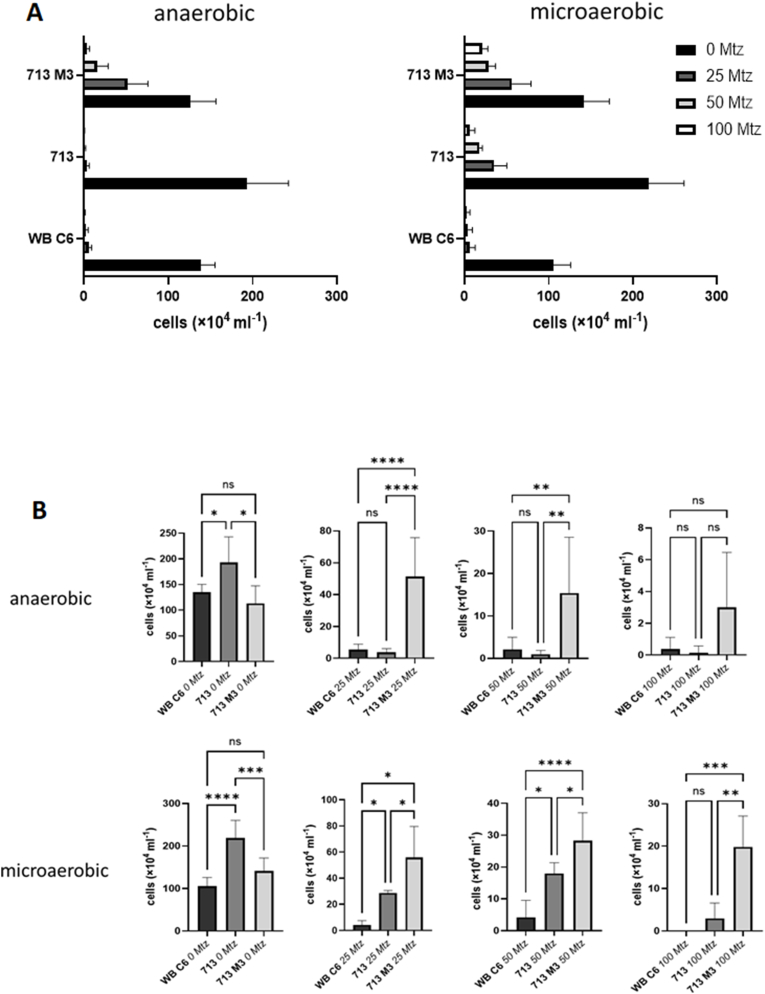
**A**, cultures of WB C6, 713, and 713-M3 were inoculated with 5 × 10^4^ cells/mL and grown at 37 °C for 48 h in the absence or presence of different concentrations of metronidazole under anaerobic or microaerophilic conditions, respectively. After 48 h cell counts were determined under the microscope. The measurements in all three strains and at all concentrations were performed independently at least five times. All metronidazole concentrations are given in μM. **B**, statistical analysis of the obtained values. A one-way ANOVA and a post-hoc Tukey test was performed with the obtained cell counts at each of the indicated metronidazole concentrations, either under anaerobic or microaerobic conditions. ns, not significant; ∗, p < 0.05; ∗∗, p < 0.01; ∗∗∗; p < 0.001; ∗∗∗∗, p < 0.0001.

**Fig. 2:**
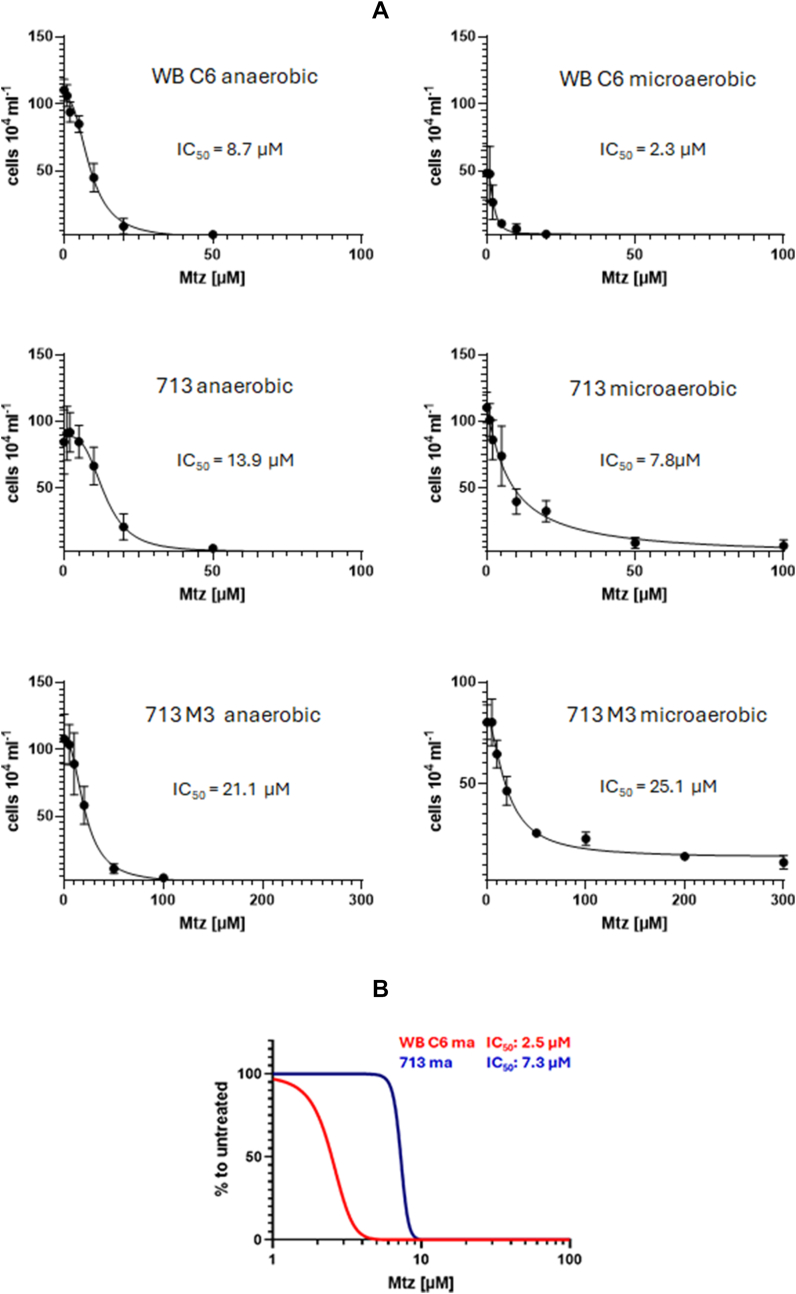
**A,** absolute IC_50_ values of metronidazole and the corresponding regression curves of WB C6, 713, and 713-M3 under anaerobic and microaerobic conditions. Assays were performed as described in Fig. 1 but inoculums for the assays were 2 × 10^4^/mL. At least four biological replicates in technical duplicates were performed. The metronidazole concentrations applied were 0, 1, 2, 5, 10, 20, 50, 100 μM metronidazole for WB C6 and 713; and 0, 5, 10, 20, 50, 100, 200, 300 μM metronidazole for 713-M3. The corresponding statistical tests for each indicated metronidazole concentration are shown and described in supplementary Fig. 1 B, normalized IC_50_ values of metronidazole in WB C6 and 713 under microaerobic conditions. The regression curves were calculated using the following setting: “[Inhibitor] vs. normalized response -- Variable slope”. The corresponding statistical tests for each indicated metronidazole concentration are shown and described in Supplementary Fig. 1. The x-axis intersects the y-axis at y = 2, equalling the inoculum (2 × 10^4^/mL), so that only values indicating growth are visually represented.

In order to test if oxygen concentrations affect the susceptibility of *G*. *lamblia* to metronidazole, cells of strain WB C6 and 713, and of a metronidazole-resistant cell line of 713, 713-M3 (Townson et al., 1992), were used for metronidazole susceptibility assays under anaerobic and microaerobic conditions. In all three strains, growth was hardly, if at all affected by oxygen when no metronidazole was added (Fig. 1). When metronidazole was added, however, strain 713 displayed a higher tolerance to metronidazole under microaerobic as compared to anaerobic conditions, with still sizeable growth at a metronidazole concentration of 25 μM (Fig. 1). Some growth could even be observed at a metronidazole concentration of 50 μM (Fig. 1). In 713-M3 growth was even observed at a metronidazole concentration of 100 μM under microaerobic conditions (Fig. 1). Interestingly, this effect was not observed in strain WB C6. In order to elucidate this protective effect of oxygen in 713 and 713-M3, the IC_50_ values of metronidazole in the three strains were determined (Fig. 2A). The determined IC_50_ values were well comparable to previously published values (Ansell et al., 2017; Hahn et al., 2013), underscoring the validity and robustness of our assay. Interestingly, the IC_50_ of metronidazole was not significantly elevated under microaerobic conditions in 713-M3, and even decreased in 713 (Fig. 2A). However, the curves obtained in microaerobic and anaerobic assays were differently shaped and a significant larger number of cells survived metronidazole treatment at higher concentrations in both strains. In WB C6 metronidazole toxicity was sharply exacerbated in the presence of oxygen (Fig. 2A), leading to a clearly lower IC_50_. We also calculated and compared the IC_50_ values of WB C6 and 713 under microaerobic conditions using normalized data (i.e. cell counts at various metronidazole concentrations normalized to cell counts of controls without the drug) because growth rates under microaerobic conditions were much lower in WB C6 than in 713, rendering absolute cell counts less significant. However, also when cell counts were normalized, the IC_50_ of metronidazole was clearly lower in WB C6 than in 713 under microaerobic conditions (Fig. 2B).

### 3.2 Growth assays with WB C6, 713, and 713-M3

**Fig. 3:**
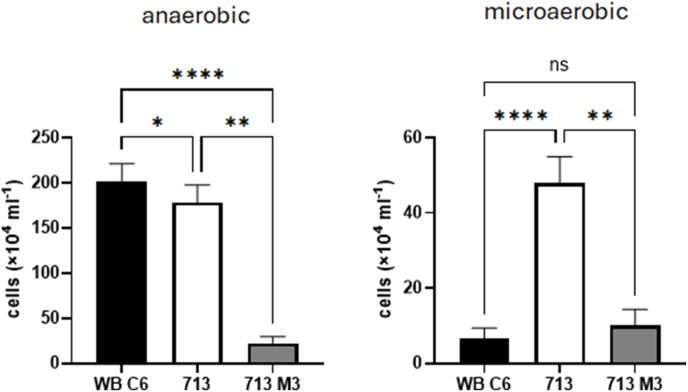
Growth of strains WB C6, 713, and 713-M3 under anaerobic and microaerophilic conditions. Inoculum size was 1 × 10^4^ cells/mL. At least four biological replicates in technical triplicates were performed with each. A one-way ANOVA and a post-hoc Tukey test was performed with the obtained cell counts. ns, not significant; ∗, p < 0.05; ∗∗, p < 0.01; ∗∗∗; p < 0.001; ∗∗∗∗, p < 0.0001.

We hypothesized that the observed effect might be caused by an overall higher fitness of 713 and 713-M3 in the presence of oxygen and performed a growth assay using smaller inoculums (2 × 10^4^ cells instead of 1 × 10^5^) in order to increase the oxygen:cell ratio. Indeed, 713 was found to grow much faster under microaerobic conditions than WB C6 (Fig. 3). Under anaerobic conditions the situation was reversed, if less pronouncedly so. Under microaerobic conditions, no significant difference between metronidazole-resistant 713-M3 and WB C6 could be observed, but under anaerobic conditions 713-M3 grew much slower than either of the two other strains.

### 3.3 Overexpression of antioxidant enzymes in 713 but not in WB C6 under microaerophilic conditions

**Fig. 4:**
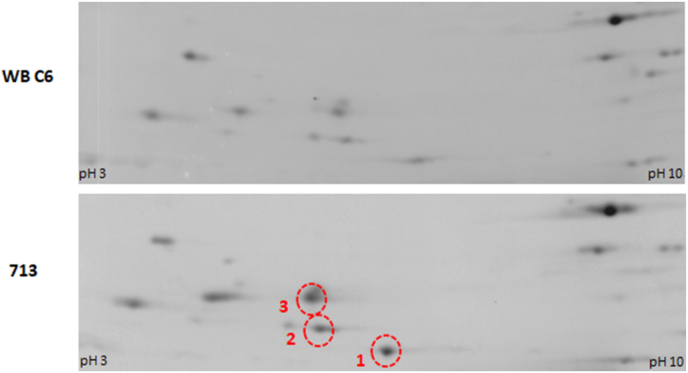
Sections of 2D gels showing upregulation of three proteins under microaerobic conditions in 713 but not in WB C6. 1., superoxide reductase (XP_037901550); 2., pyridoxamine 5′-phosphate oxidase putative domain-containing protein (XP_001705592); 3., TlpA-like family protein (XP_001707531). Gels show a pH range of 3–10; 12.5% PAA. The complete images are shown in Supplementary Fig. 2.

Next, we wanted to assess if this observed differential performance in the presence of oxygen was caused by differential expression of antioxidant enzymes in the two strains. To this end we grew 713 and WB C6 under anaerobic and microaerobic conditions for 48 h in plates as done before for the susceptibility assays, harvested the cells, and compared the protein expression profiles by two-dimensional gel electrophoresis. We identified three proteins which were clearly upregulated in expression under microaerobic conditions in 713 but not in WB C6 (Fig. 4). Densitometric analyses indicated a threefold overexpression of all three proteins in 713 as compared to WB C6 (Table 1). The protein spots were excised and analyzed by mass spectrometry. The following proteins were identified as the major components of the respective protein spots (Supplementary Table 2): 1., superoxide reductase (XP_037901550); 2., a pyridoxamine 5′-phosphate oxidase putative domain-containing protein (XP_001705592); and 3., a TlpA-like family protein (XP_001707531). Superoxide reductase is a well characterized antioxidant factor (Testa et al., 2011), and TlpA is thioredoxin-like protein which is also consistent with the observed upregulation under microaerobic conditions. Finally, according to the InterPro database pyridoxamine 5-phosphate oxidase is predicted to contain an FMN-binding domain which is also indicative of an antioxidant function.

**Table 1:** Protein expression levels of antioxidant enzymes in WB C6 and 713 under microaerobic conditions.

Number	Description	NCBI entry	WB C6 (%Vol)	713 (%Vol)
1	Superoxide reductase	XP_037901550	0.2	0.6
2	Pyridoxamine 5′-phosphate oxidase	XP_001705592	0.2	0.4
3	TlpA	XP_001707531	0.5	1.4

### 3.4 *Episomal overexpression of the antioxidant enzymes in* G. lamblia *WB C6 and assessment of the effect on oxygen tolerance and metronidazole susceptibility*

**Fig. 5:**
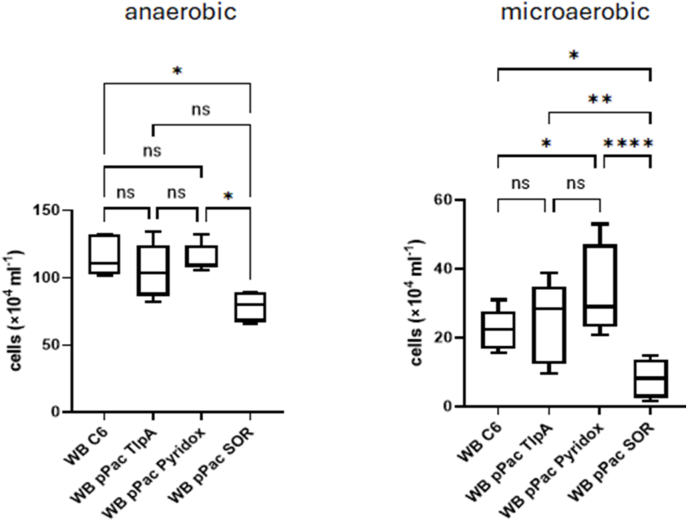
Growth of strain WB C6 and the transfected cell lines WB pPac Pyridox, WB pPac TlpA, and WB pPac SOR under anaerobic and microaerophilic conditions. Inoculum size was 1 × 10^4^ cells/mL. ∗, p < 0.05; ∗∗, p < 0.01; ∗∗∗; p < 0.001; ∗∗∗∗, p < 0.0001 according to a one-way ANOVA with subsequent Tukey test. Cell counts were performed at least in 4 biological replicates in technical duplicates.

We wanted to test if episomal overexpression of either one of the three identified enzymes would confer higher tolerance to oxygen and/or metronidazole in WB C6 and attempted to generate the respective transfected cell lines using the pPac-VInteg vector (Štefanić et al., 2009). Pyridoxamine 5-phosphate oxidase and TlpA could be overexpressed in WB C6 to a large extent (Fig. 5) using the very strong arginine deiminase (ADI) promoter, as done in a previous study (Leitsch et al., 2016). Overexpression of superoxide reductase using the ADI promoter, however, proved to be toxic to the cells as no survivors could be salvaged after transfection with the respective plasmid, pPac SOR. When the ADI promoter was exchanged for the weaker glutamate dehydrogenase (GDH) promoter (Müller et al., 2009), superoxide reductase could be strongly overexpressed (Supplementary Fig. 3) in WB C6. When we tested the respective cell lines, WB pPac Pyridox, WB pPac TlpA and WB pPac SOR for oxygen tolerance, we found that overexpression of pyridoxamine 5-phosphate oxidase did indeed enhance growth in the presence of oxygen whereas overexpression of TlpA did not (Fig. 5). In contrast, overexpression of superoxide reductase rendered cells highly susceptible to oxygen, presumably because the enzyme produces hydrogen peroxide (Fig. 5).

**Fig. 6:**
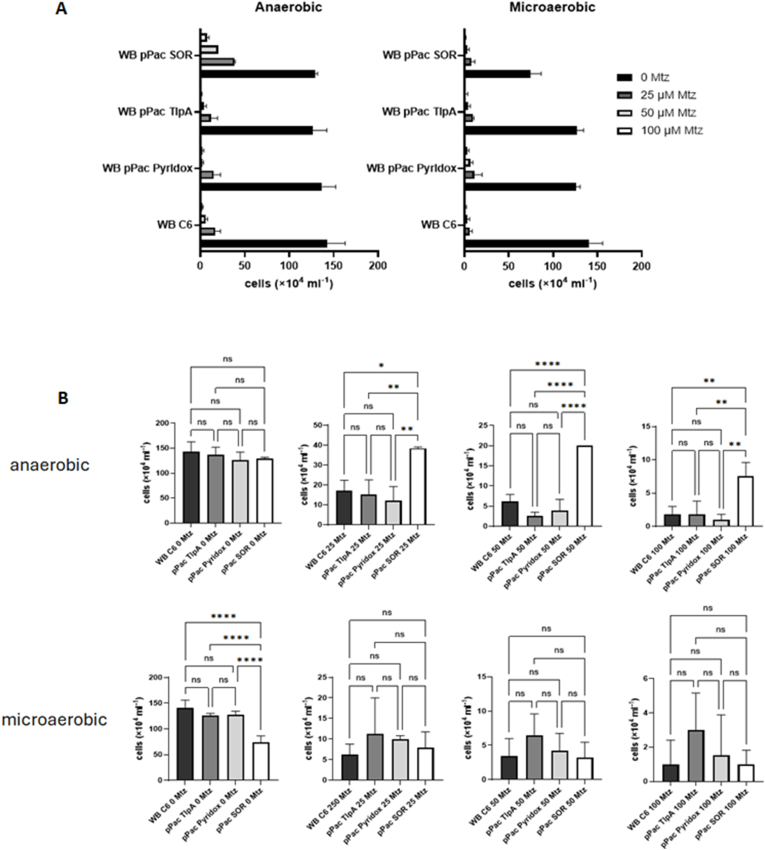
**A**, cultures of WB C6, WB pPac Pyridox, WB pPac TlpA, and WB pPAc SOR were inoculated with 5 × 10^4^ cells/mL and grown at 37 °C for 48 h in the absence or presence of different concentrations of metronidazole under anaerobic or microaerophilic conditions, respectively. After 48 h cell counts were determined under the microscope. The measurements in all four strains and at all concentrations were performed independently at least four times. All metronidazole concentrations are given in μM. **B**, statistical analysis of the obtained values. A one-way ANOVA and a post-hoc Tukey test was performed with the obtained cell counts at each of the indicated metronidazole concentrations, either under anaerobic or microaerobic conditions. ns, not significant; ∗, p < 0.05; ∗∗, p < 0.01; ∗∗∗; p < 0.001; ∗∗∗∗, p < 0.0001.

**Fig. 7:**
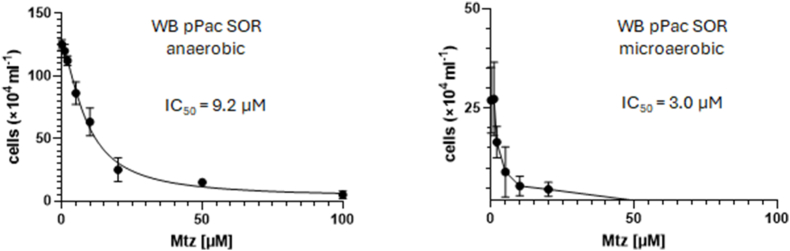
Absolute IC_50_ values of metronidazole and the corresponding regression curves of WB pPac SOR under anaerobic and microaerobic conditions. Assays were performed as described in Fig. 1 but inoculums for the assays were 2 × 10^4^ cells/mL. At least four biological replicates in technical duplicates were performed. The metronidazole concentrations applied were 0, 1, 2, 5, 10, 20, 50, 100 μM metronidazole. The corresponding statistical tests for each indicated metronidazole concentration are shown and described in Supplementary Fig. 1. The x-axis intersects the y-axis at y = 2, equalling the inoculum (2 × 10^4^/mL), so that only values indicating growth are visually represented.

When we tested the impact of the antioxidant enzymes on the susceptibility to metronidazole, we found neither a positive nor a negative effect of pyridoxamine 5-phosphate oxidase and TlpA as compared to control (Fig. 6). Superoxide reductase, however, provided a clearly protective effect against metronidazole under anaerobic but not microaerobic conditions (Fig. 6). When we determined the IC_50_ of metronidazole for WB pPac SOR (Fig. 7), we obtained a curve very well comparable to that of strain 713 under microaerophilic conditions (Fig. 2), suggesting that the observed protective effect against metronidazole might indeed be conferred by increased superoxide reductase expression. In contrast, the IC_50_ of metronidazole in WB pPac SOR under microaerophilic conditions was as low as in the parent strain WB C6 (Fig. 2), arguably because elevated intracellular hydrogen peroxide levels caused by elevated superoxide reductase activity countermanded the protective effect against metronidazole.

### 3.5 Clinical isolate P064 from a refractory case displays elevated tolerance to metronidazole under microaerobic conditions similarly to 713

**Fig. 8:**
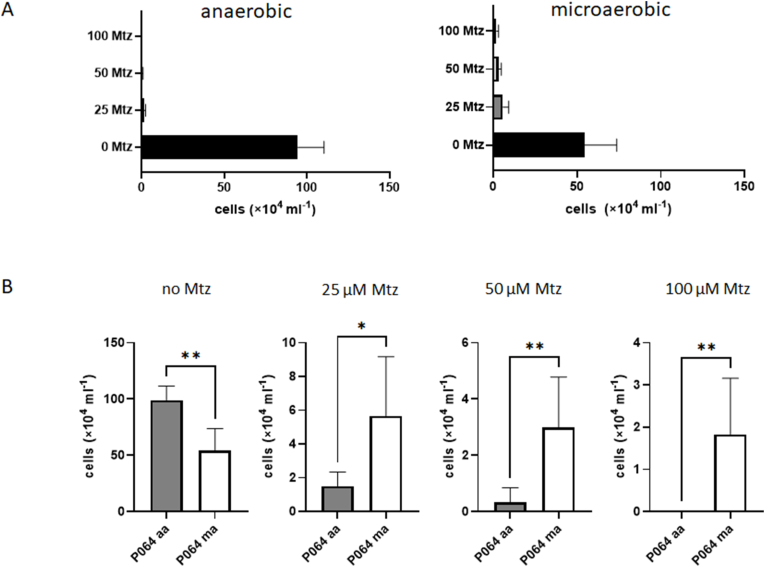
**A**, cultures of P064 were inoculated with 5 × 10^4^ cells/mL and grown at 37 °C for 48 h in the absence or presence of different concentrations of metronidazole under anaerobic or microaerophilic conditions, respectively. After 48 h cell counts were determined under the microscope. The measurements in all three strains and at all concentrations were performed independently at least four times. All metronidazole concentrations are given in μM. **B**, statistical analysis of the obtained values. T tests were performed with the obtained cell counts at each of the indicated metronidazole concentrations, either under anaerobic or microaerobic conditions. ns, not significant; ∗, p < 0.05; ∗∗, p < 0.01.

**Fig. 9:**
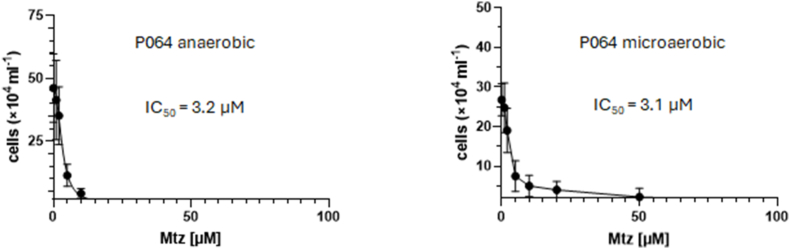
Absolute IC_50_ values of metronidazole and the corresponding regression curves of P064 under anaerobic and microaerobic conditions. Assays were performed as described in Fig. 1 but inoculums for the assays were 2 × 10^4^ cells/mL. At least four biological replicates in technical duplicates were performed. The metronidazole concentrations applied were 0, 1, 2, 5, 10, 20, 50, 100 μM metronidazole. The corresponding statistical tests for each indicated metronidazole concentration are shown and described in Supplementary Fig. 1. The x-axis intersects the y-axis at y = 2, equalling the inoculum (2 × 10^4^/mL), so that only values indicating growth are visually represented.

As a last step we wanted to assess if the protective effect of oxygen against metronidazole could be of relevance in clinical *G*. *lamblia* isolates. To that end, we tested two clinical strains, P064 and P344 (Klotz et al., 2023), from the Robert Koch-Institute in Berlin in our experimental setup. Importantly, P064 (then designated as “14-03/F7”) had been isolated from a giardiasis patient refractory to metronidazole treatment (Hahn et al., 2013). Unfortunately, strain P344 (assemblage BIII) did not grow in the micro well plates, regardless of the oxygen concentrations applied. P064, however, did grow in the micro well plates, if clearly slower than the established laboratory strains WB C6 and 713. When we determined the susceptibility to metronidazole under anaerobic and microaerophilic conditions in P064, we observed an overall higher susceptibility of this strain to metronidazole than with WB C6 and 713 (Fig. 8). This had been observed before (Hahn et al., 2013). Still, in the presence of oxygen more surviving cells could be observed at higher metronidazole concentrations than under anaerobic conditions, mirroring the situation in strain 713 (Fig. 1). Again, the IC_50_ of metronidazole was not elevated under microaerobic concentrations (Fig. 9), but the curve was differently shaped as observed previously in 713 (Fig. 2).

## 4 Discussion

In this study we demonstrate that oxygen concentrations affect the susceptibility to metronidazole in *G*. *lamblia*. In strain 713 the susceptibility to metronidazole was clearly decreased under microaerobic conditions as generated by CampyGen (Fig. 1, Fig. 2). A similar observation was made in the metronidazole-resistant derivative strain of 713, 713-M3 (Fig. 1, Fig. 2). We do not understand this protective effect as resistance in the strict sense, but as an enhanced survival rate of individual cells at high metronidazole concentrations close to the reported serum levels of metronidazole (Ralph et al., 1974). In contrast to the truly resistant cell line 713 M3, which displays a clearly higher IC_50_ of metronidazole as compared to parental 713 (Fig. 2A), this effect is not, or in some instances only slightly, reflected by the determined IC_50_ values (Fig. 2, Fig. 6, Fig. 9).

The CampyGen sachets were specifically developed for the culture of bacteria colonizing the small intestine. Since *G*. *lamblia* colonizes the same niche, it is safe to assume that the microaerobic atmosphere generated by CampyGen is very close to the situation which *G*. *lamblia* encounters in the host. The oxygen-related effect on metronidazole susceptibility observed in strain 713, but not in strain WB C6 (Fig. 1, Fig. 2), was clearly less pronounced than in metrondazole-resistant clinical *Trichomonas vaginalis* isolates (Meingassner and Thurner, 1979), but it could affect the treatment outcome nevertheless. Strain 713 also displayed an overall higher fitness in the presence of elevated oxygen concentrations than WB C6 (Fig. 3). This higher tolerance to oxygen was not observed in the metronidazole-resistant cell line of 713, 713-M3 (Townson et al., 1992). However, this strain was generated by mutagenesis with UV light and cannot infect mice, whereas parental 713 can (Tejman-Yarden et al., 2011). Further, it displays certain impairments as compared to 713, such as a strongly decreased NADPH oxidase/flavin reductase activity (Leitsch et al., 2011) and impaired adherence to epithelial cells (Tejman-Yarden et al., 2011). The analogous enzyme activity is known to be negatively associated metronidazole resistance in *T*. *vaginalis* (Leitsch et al; Mayr et al., 2024). Thus, metronidazole tolerance in this strain is likely to be caused by other, yet unresolved mechanisms and not only by the mere overexpression of antioxidant enzymes. Still, also 713-M3 was clearly less susceptible to metronidazole under microaerobic conditions (Fig. 1, Fig. 2).

In a clinical isolate from a refractory case, P064 (Hahn et al., 2013), susceptibility to metronidazole was also reduced under microaerobic conditions similarly to the situation in 713. However, since this strain performed clearly worse in our assay than both laboratory strains WB C6 and 713 (Fig. 8, Fig. 9), it is premature to draw firm conclusions. First, assay conditions have to be optimized in order to receive reliable results with clinical isolates. This is even more obvious with the other isolate we tested, P344, which did not grow at all in the micro well plates we used for the assays. Together with the difficulties of obtaining axenic cultures of *Giardia* isolates in the first place, inadequate testing methods have hampered an understanding of metronidazole resistance in clinical isolates so far. Given the steep increase of metronidazole resistance in *G*. *lamblia* in the last decade (Muñoz Gutierrez et al., 2013; Nabarro et al., 2015), however, it is certainly pressing to pursue this line of research.

In addition to an improved tolerance to metronidazole in 713 under microaerobic conditions, we also observed that 713 grows much faster under microaerobic conditions than WB C6 (Fig. 3). Interestingly, this was accompanied by the overexpression of three enzymes, i.e. pyridoxamine 5-phosphate oxidase, TlpA, and SOR, (Fig. 4). In WB C6 no such overexpression was observed. Of these proteins, superoxide reductase has already been characterized (Testa et al., 2011). Pyridoxamine 5-phosphate oxidase has not been characterized but it was found to be secreted (Dubourg et al., 2018) and the corresponding gene was identified as being one of the most strongly expressed in *G*. *lamblia* (Ma'ayeh et al., 2015). TlpA is a novel protein in *G*. *lamblia* but resembles thioredoxins. The overexpression of pyridoxamine 5-phosphate oxidase did indeed improve growth of WB C6 under microaerobic conditions (Fig. 5), but did not increase tolerance to metronidazole (Fig. 7). In contrast, the overexpression of superoxide reductase rendered WB C6 clearly more sensitive to oxygen but more tolerant to metronidazole (Fig. 6, Fig. 7), albeit only under anaerobic rather than microaerobic conditions. Presently, it is unclear if superoxide reductase interacts directly with metronidazole, or if the protective effect is conferred indirectly by affecting the activities of other enzymes. Superoxide reductase, alternatively termed desulfoferredoxin, might be able to reduce the nitro group of metronidazole, but the reduction of metronidazole is commonly understood as a necessary step to render the drug toxic. On the other hand, Nim proteins, which occur in anaerobic bacteria such as *Bacteroides* spp. and *Clostridioides difficile*, have been proposed to act as nitroreductases which transfer 6 electrons to metronidazole's nitro group in order to form a non-toxic aminoimidazole (reviewed in Leitsch, 2019). In any case, the strong overexpression of superoxide reductase alone is problematic in the presence of oxygen because it produces hydrogen peroxide. In fact, *Giardia* is highly sensitive to hydrogen peroxide and already very low concentrations of 50 μM–100 μM can kill trophozoites rapidly (Hill and Pearson, 1987). In contrast, *Entamoeba histolytica* easily survives hydrogen peroxide concentrations higher than 1 mM (Ghosh et al., 2010). Importantly, hydrogen peroxide quickly disables oxygen scavenging mechanisms in *Giardia* (Mastronicola et al., 2011), rendering the parasite defenceless against oxidative stress. Thus, the protective effect against metronidazole as conferred by superoxide reductase is probably countermanded in the presence of oxygen. Accordingly, a concomitant upregulation of other antioxidant enzymes which either remove intracellular oxygen before it can be reduced to hydrogen peroxide, or remove hydrogen peroxide, respectively, might be necessary. At least pyridoxamine 5-phosphate oxidase is likely to exert such antioxidant function as it improved growth in WB C6 under microaerobic conditions upon overexpression (Fig. 6). Further work, however, is necessary to identify its actual function. TlpA, if indeed functioning as a thioredoxin, might indirectly enhance removal of hydrogen peroxide by reducing peroxiredoxin (Mastronicola et al., 2014). Importantly, *G*. *lamblia* does not have catalase but instead relies on peroxiredoxin for the removal of hydrogen peroxide. In addition, other factors which were not identified in this study are likely to be involved in mediating enhanced oxygen and metronidazole tolerance. 2D gel electrophoresis is a convenient method and allows immediate comparisons of expression levels of intact proteins, but due to sensitivity issues it misses many, if not the majority of the proteins expressed. Thus, more sophisticated approaches such as shotgun mass spectrometric analysis based on multiplex tandem mass tags will provide a larger number of candidate proteins.

As a conclusion, it is interesting to note that the herein described culture of *G*. *lamblia* under microaerobic conditions might prove a helpful tool also in the study of scientific questions other than metronidazole resistance. *Giardia lamblia* is essentially a microaerophilic and not an anaerobic parasite, and many scientific questions could be more adequately addressed when applying growth conditions resembling the *in vivo* situation.

## CRediT authorship contribution statement

**Kateryna Starynets:** Investigation. **Ana Paunkov:** Methodology, Investigation. **Anja Wagner:** Writing – original draft, Investigation. **Klaus Kratochwill:** Writing – review & editing, Validation, Methodology. **Christian Klotz:** Resources, Formal analysis. **David Leitsch:** Writing – original draft, Validation, Supervision, Resources, Project administration, Methodology, Investigation, Funding acquisition, Formal analysis, Data curation, Conceptualization.

## Declaration of competing interest

We declare that there is no conflict of interest whatsoever pertinent to this study.
