# Supplementary material for: Culturing of Giardia lamblia under microaerobic conditions can impact metronidazole susceptibility by inducing increased expression of antioxidant enzymes
Source: Int J Parasitol Drugs Drug Resist. 2025 Feb 1;27:100585. doi: 10.1016/j.ijpddr.2025.100585 (PMC11847123; doi:10.1016/j.ijpddr.2025.100585)
Supplement: Multimedia component 1 [file mmc1.pdf]

Supplementary Figure 1: Statistical analysis of cell counts used for the calculation of IC_{50} curves

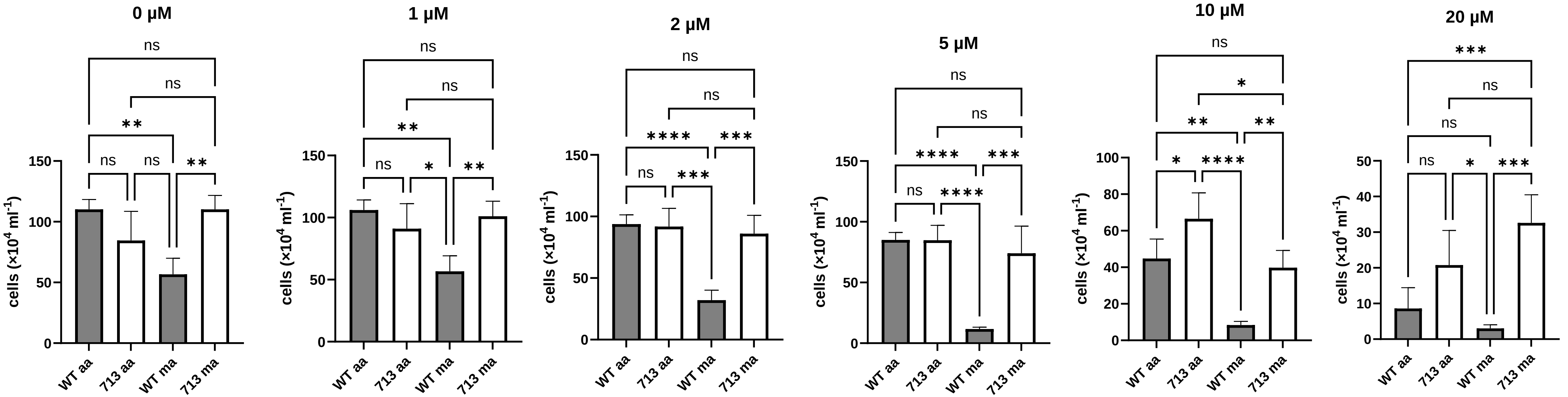

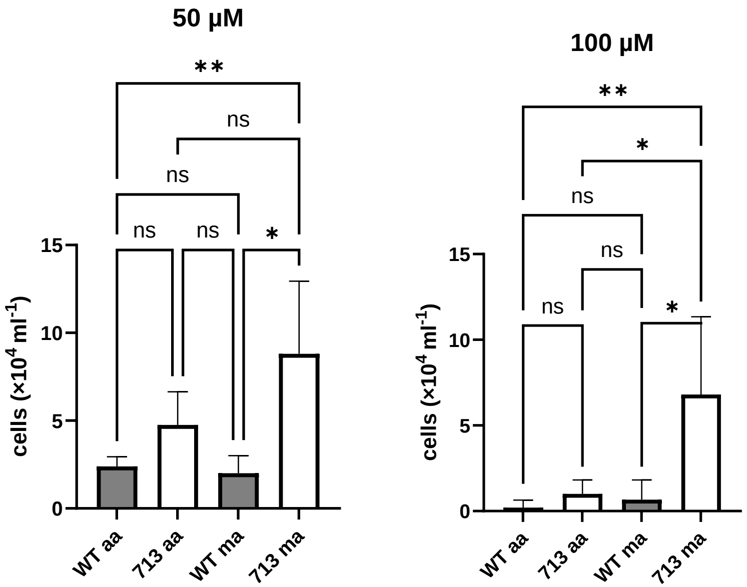

See Figure 2A: IC_{50} calculations for metronidazole with strains WB C6 (WT) and 713 under anaerobic and microaerobic conditions. Values at a given concentration were tested for significance by applying a one-way ANOVA and a post-hoc Tukey test.

ns	not significant
*	$p < 0.05$
**	$p < 0.01$
***	$p < 0.001$
****	$p < 0.0001$

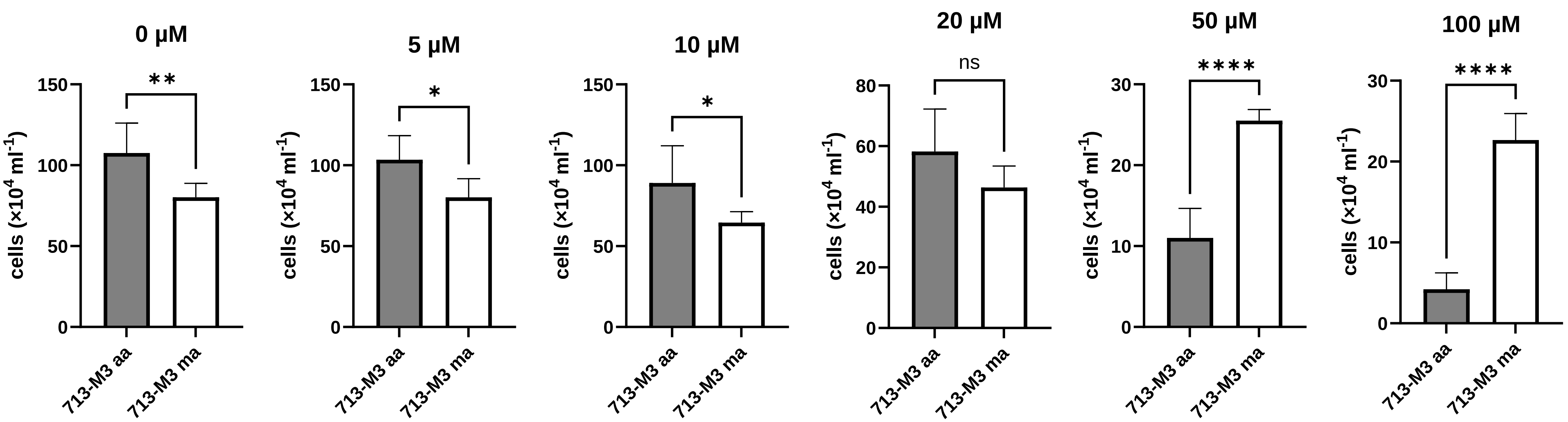

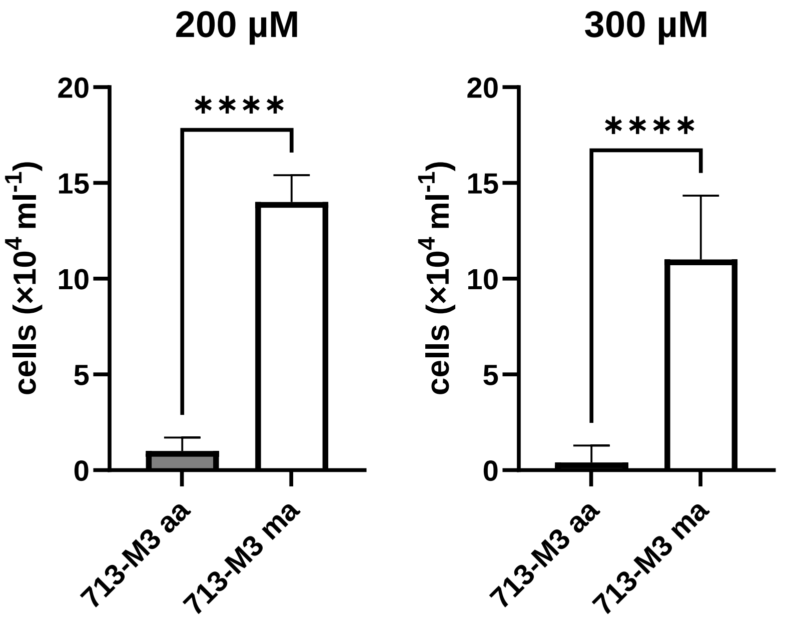

See Figure 2A: IC_{50} calculations for metronidazole with strain 713-M3 under anaerobic and microaerobic conditions. Values at a given concentration were tested for significance by applying an unpaired t-test.

ns	not significant
*	$p < 0.05$
**	$p < 0.01$
***	$p < 0.001$
****	$p < 0.0001$

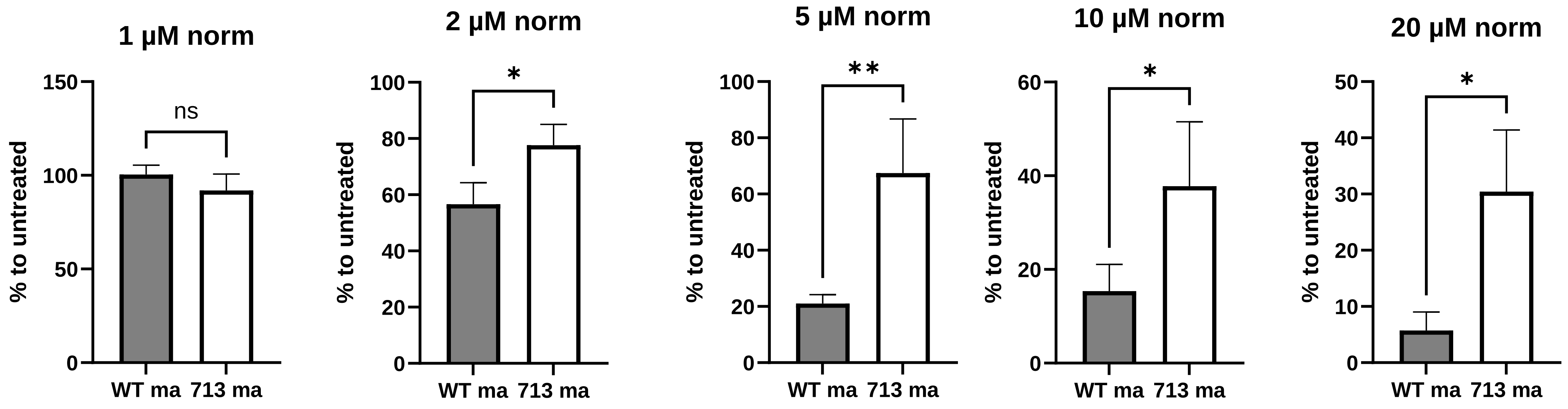

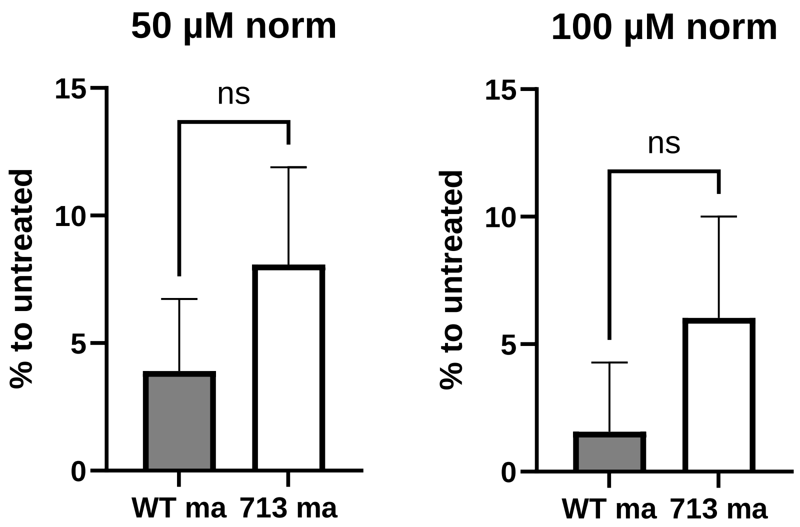

See Figure 2B: IC_{50} calculations for metronidazole under microaerobic conditions, normalized to the respective untreated controls with strains WB C6 (WT) and 713. Values at a given concentration were tested for significance by applying an unpaired t test.

ns	not significant
*	$p < 0.05$
**	$p < 0.01$

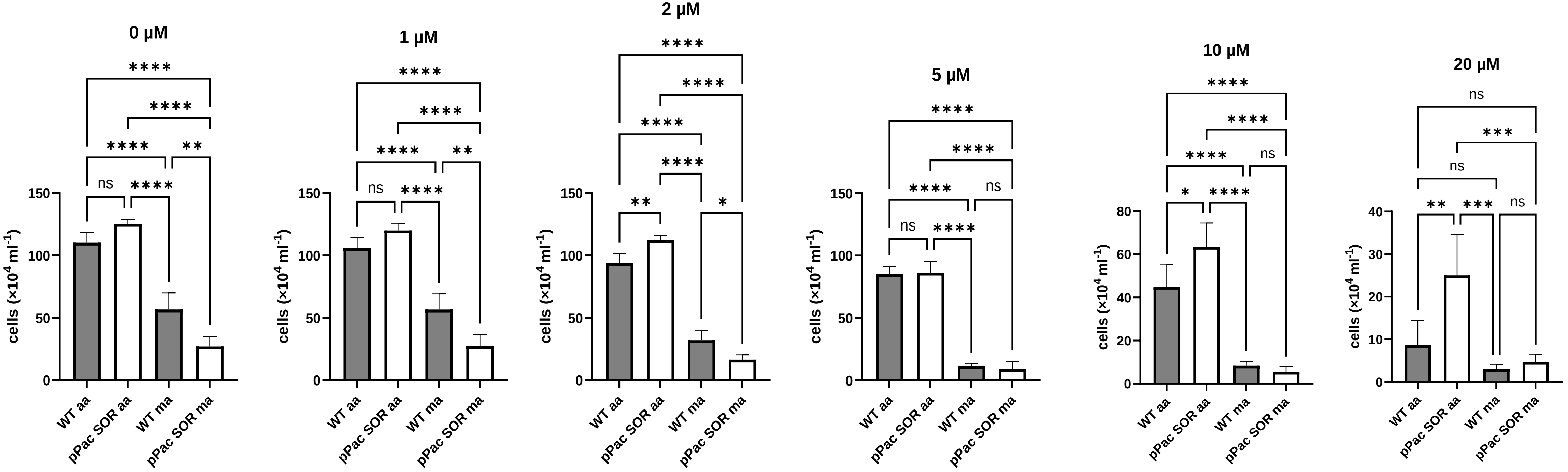

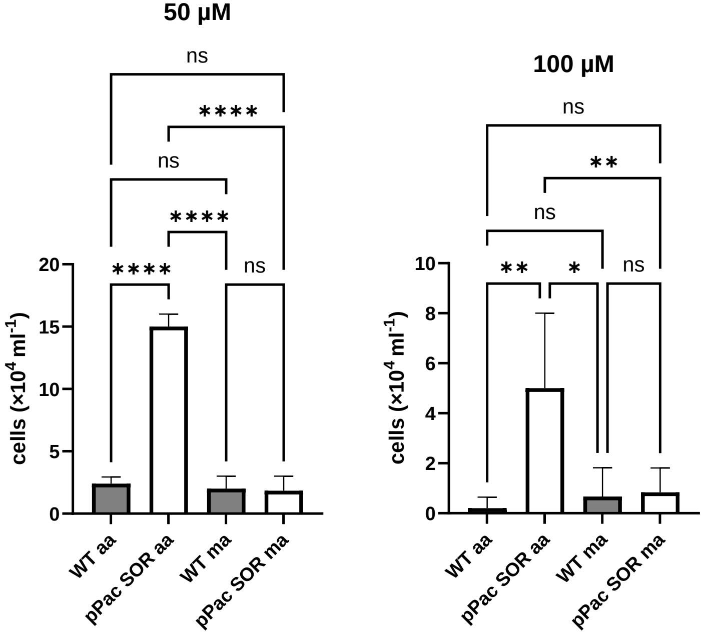

See Figure 7: IC_{50} calculations for metronidazole with strains WB C6 (WT) and WB pPac SOR under anaerobic and microaerobic conditions. Values at a given concentration were tested for significance by applying a one-way ANOVA and a post-hoc Tukey test.

ns	not significant
*	$p < 0.05$
**	$p < 0.01$
***	$p < 0.001$
****	$p < 0.0001$

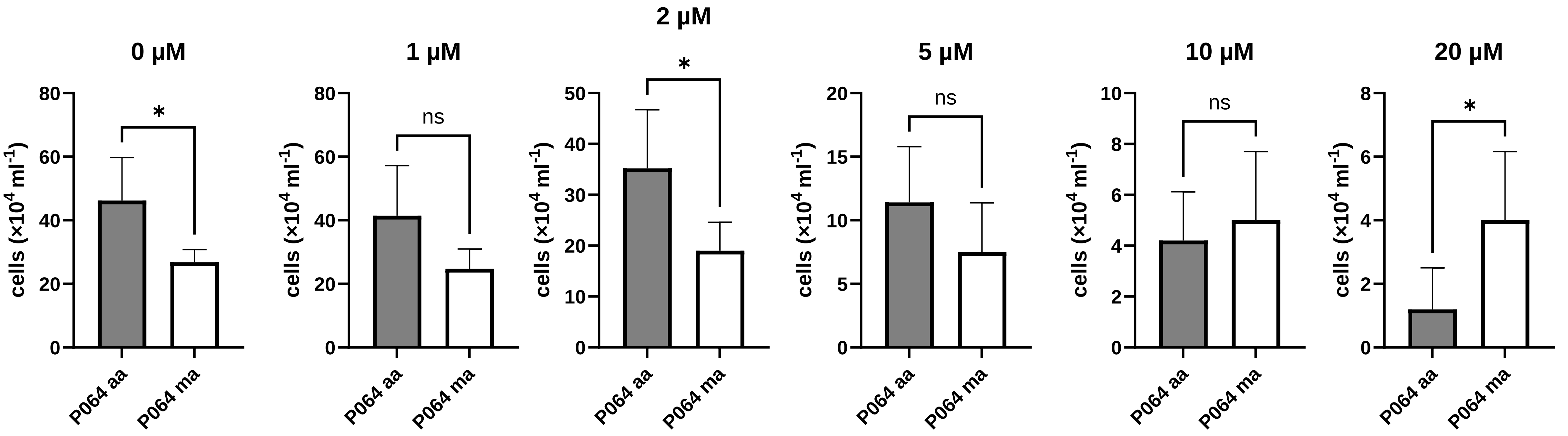

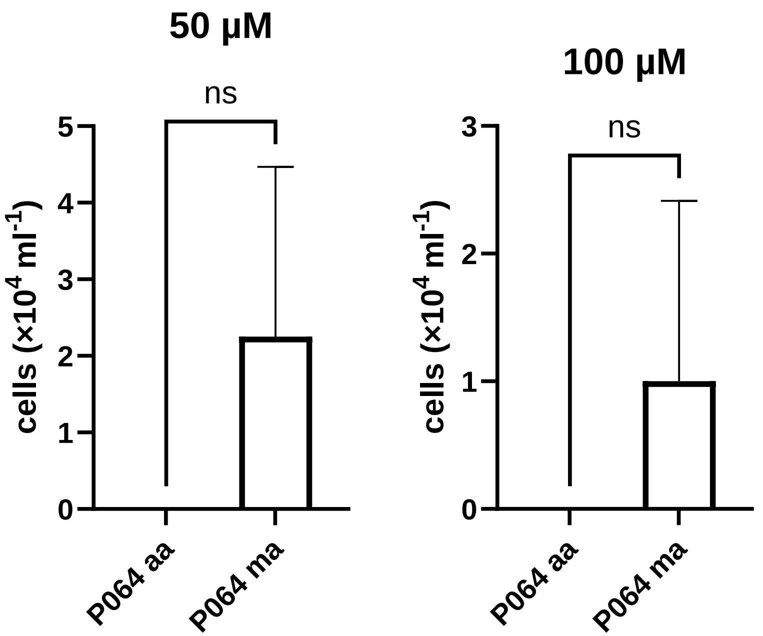

See Figure 9: IC_{50} calculations for metronidazole with strain P064 under anaerobic and microaerobic conditions. Values at a given concentration were tested for significance by applying an unpaired t test.

ns   not significant
* $p < 0.05$
