# Supplementary material for: Culturing of Giardia lamblia under microaerobic conditions can impact metronidazole susceptibility by inducing increased expression of antioxidant enzymes
Source: Int J Parasitol Drugs Drug Resist. 2025 Feb 1;27:100585. doi: 10.1016/j.ijpddr.2025.100585 (PMC11847123; doi:10.1016/j.ijpddr.2025.100585)
Supplement: Multimedia component 2 [file mmc2.pdf]

Comparison of 2D profiles in WB C6 and 713 under microaerophilic conditions

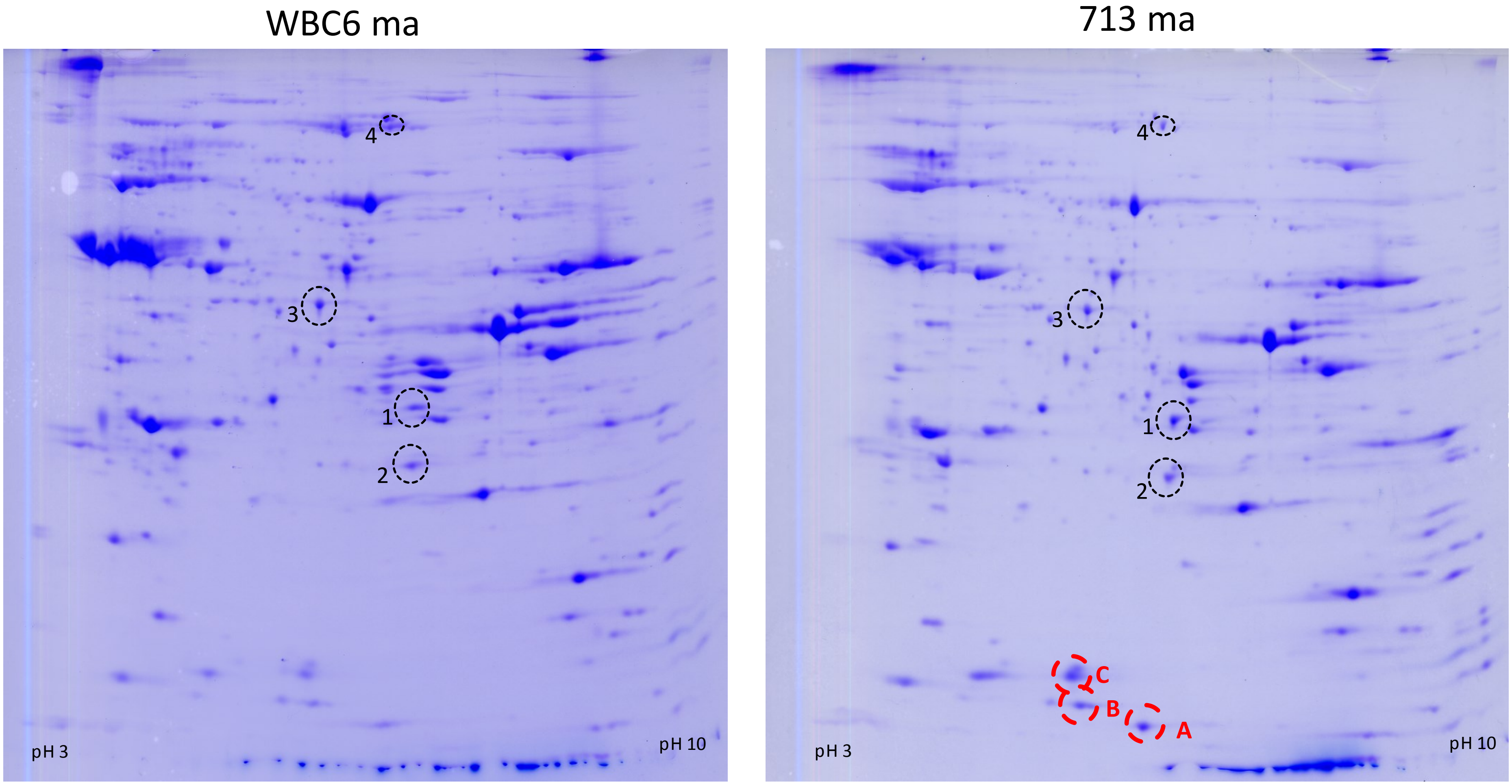

PAA: 12.5%
pH range: 3 – 10 (non-linear)

A., superoxide reductase (XP_037901550)
B., pyridoxamine 5'-phosphate oxidase putative domain-containing protein (XP_001705592)
C., TlpA-like family protein (XP_001707531).

For comparison, some proteins identified in an earlier study (Leitsch et al., 2012; doi: 10.1016/j.ijpddr.2012.04.002) are highlighted:

1: thioredoxin reductase (34 kDa)
2: *Giardia* trophozoite antigen 2 (26 kDa)
3: branched chain amino-transferase (39 kDa)
4: alcohol dyhydrogeanse (97 kDa)
