# Supplementary material for: Culturing of Giardia lamblia under microaerobic conditions can impact metronidazole susceptibility by inducing increased expression of antioxidant enzymes
Source: Int J Parasitol Drugs Drug Resist. 2025 Feb 1;27:100585. doi: 10.1016/j.ijpddr.2025.100585 (PMC11847123; doi:10.1016/j.ijpddr.2025.100585)
Supplement: Multimedia component 3 [file mmc3.pdf]

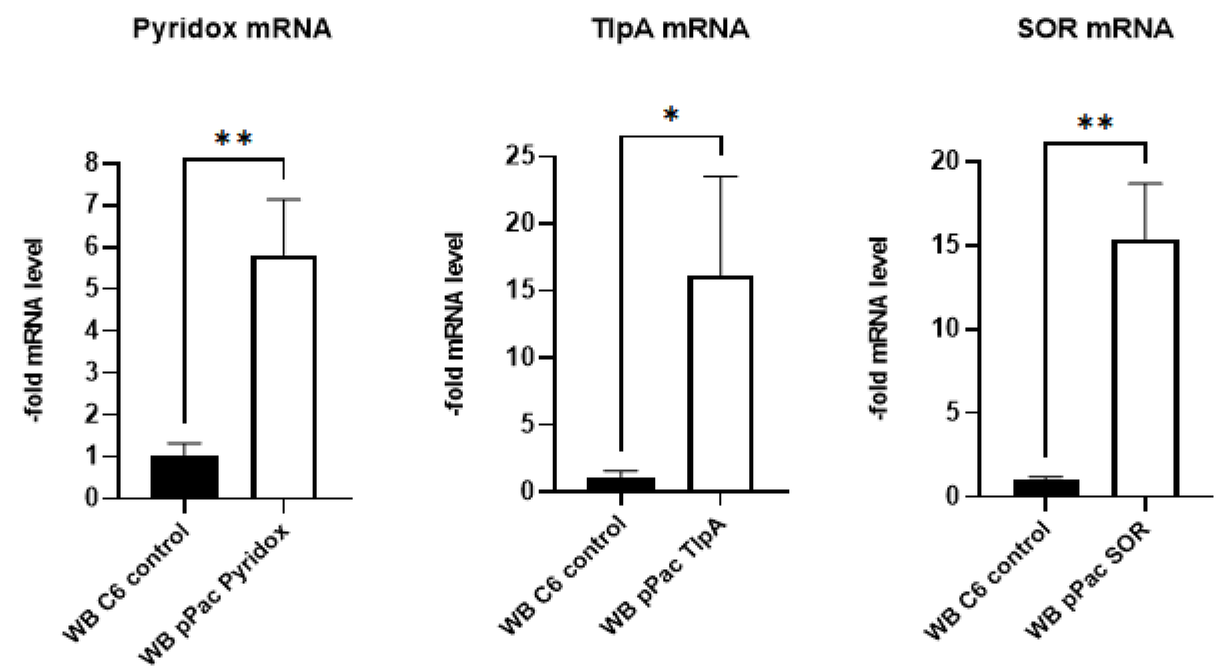

Supplementary Figure 3: RT-qPCR measurement of the relative abundance of pyridoxamine 5'-phosphate oxidase mRNA, TlpA mRNA, and superoxide reductase mRNA in WB C6 and the three transfected cell lines WB pPac Pyridox, WB pPac TlpA, and WB pPac SOR. Unpaired t tests were performed. *, $p < 0.05$; **, $p < 0.01$. Abundancies of mRNAs were determined in at least three biological replicates in technical triplicates.
